# Surgical aortic valve replacement with Y-incision aortic annular enlargement provided better hemodynamics than transcatheter aortic valve replacement

**DOI:** 10.1016/j.xjse.2025.100091

**Published:** 2025-11-25

**Authors:** Katelyn Monaghan, Marc Titsworth, Divyaam Satija, Bahar Masoudian, China Green, Aditya Sridhar, Gorav Ailawadi, Shinichi Fukuhara, Himanshu Patel, Barbara Hamilton, George Michael Deeb, Bo Yang

**Affiliations:** Department of Cardiac Surgery, Michigan Medicine, Ann Arbor, Mich

**Keywords:** SAVR, TAVR, Y-incision aortic annular enlargement, aortic root enlargement

## Abstract

**Objective:**

We aimed to examine early outcomes of surgical aortic valve replacement (SAVR) with Y-incision aortic annular enlargement (Y-AAE) versus transcatheter AVR (TAVR) in native aortic valve stenosis.

**Methods:**

From August 2020 to March 2024, 362 patients with severe native aortic valve stenosis underwent SAVR + Y-AAE with bioprosthetic valves (n = 70) or TAVR (n = 292) with ejection fraction ≥50%, Society of Thoracic Surgeons predicted risk of mortality score ≤8, and minimal aortic annular diameter ≤25 mm by computed tomography. Nearest-neighbor 1:3 propensity score matching was conducted across all preoperative variables.

**Results:**

The minimal aortic annular diameter by CT was 22 mm (IQR, 20, 23 mm) in the TAVR group and 21 mm (IQR, 19, 23 mm) in the SAVR + Y-AAE group. The TAVR group had median implanted valve sizes of Edwards LifeSciences Sapien 26 mm (IQR, 25, 29 mm) and Medtronic Evolut 29 mm (29, 34 mm), whereas the SAVR + Y-AAE group had a median implant size of 29 mm (27, 29 mm). Compared with the TAVR group, the SAVR + Y-AAE group had similar operative mortality (0% vs 2%; *P* > .99) and lower postoperative pacemaker implantation rate (1.4% vs 10.3%; *P* = .03). At 24 to 36 months postoperative, the SAVR + Y-AAE group had significantly larger effective orifice area (2.7 vs 1.9 cm^2^), lower aortic valve mean gradients (5 vs 9.5 mm Hg), greater dimensionless index (0.67 vs 0.54), lower rates of aortic insufficiency (3.0% vs 26%), and less moderate/severe prosthesis-patient mismatch (0% vs 20.1%). The left ventricular mass index regression was greater in SAVR + Y-AAE patients (42% vs 22%; *P* = .05). The SAVR + Y-AAE 3-year survival was 96% versus 79% for TAVR in the propensity-score matched cohort (*P* = .12). The hazard ratio of SAVR + Y-AAE for early mortality was 0.15 (95% CI, 0.02-1.13; *P* = .066).

**Conclusions:**

Low- and intermediate-risk patients with aortic valve stenosis should be considered for SAVR + Y-AAE for excellent hemodynamics and early outcomes.

## Graphical Abstract

**Figure:**
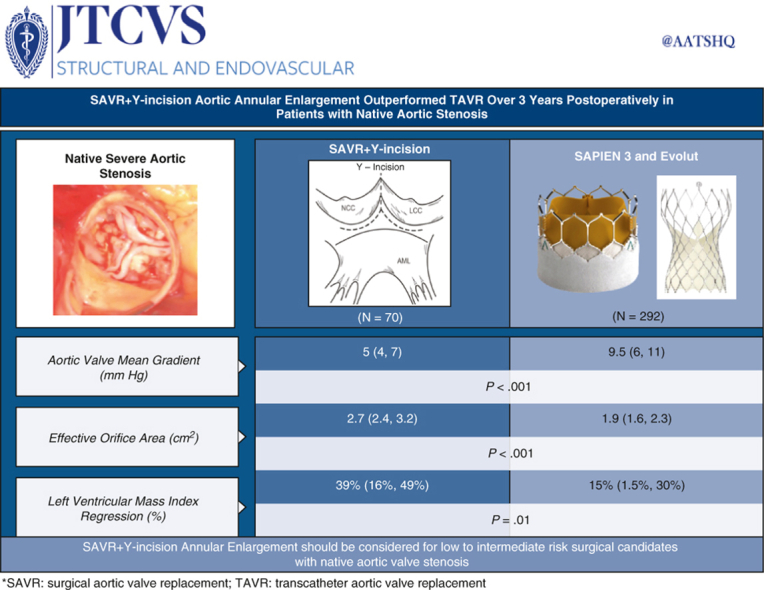

**Figure:**
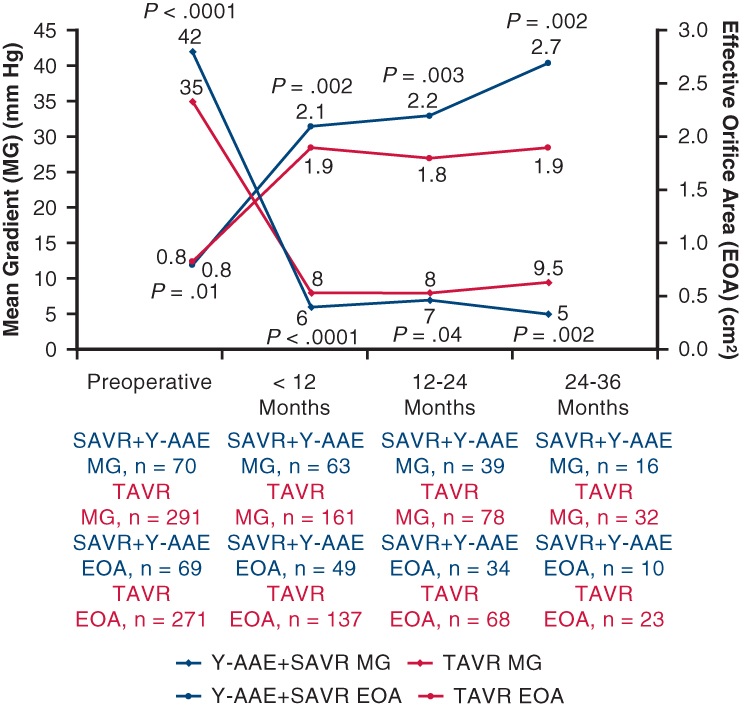
SAVR + Y-AAE provided better EOA and MG than TAVR in patients with aortic stenosis.

**Central Message**

SAVR with Y-incision aortic annular enlargement outperforms TAVR in patients with native aortic valve stenosis.

**Perspective**

SAVR with Y-AAE achieved significantly better hemodynamics than TAVR in patients with native aortic stenosis during 3-year follow-up. SAVR + Y-AAE should be considered for low- and intermediate-risk patients with aortic stenosis.

Several trials have demonstrated superior or similar hemodynamics in transcatheter aortic valve replacement (TAVR) compared with surgical aortic valve replacement (SAVR).^1^^,^^2^ Among all TAVR versus SAVR trials, the most common stented valve sizes in the SAVR cohort are sizes 21 and 23. The Placement of Aortic Transcatheter Valves 3 (PARTNER 3) trial reports the greatest rate of aortic root enlargement in the SAVR group (4.6%) among all TAVR versus SAVR trials, yet 57% of SAVR patients received a size 23 valve or smaller.^3^ Similarly, the Evolut Low-Risk trial reports aortic root enlargement in only 1.6% of patients undergoing SAVR, whereas 53% of patients undergoing SAVR received a size 23 prosthesis or smaller.^4^

As we previously reported, the Y-incision aortic annular enlargement (Y-AAE) provided a safe and effective way to upsize the prosthetic valve implanted by 3 to 4 valve sizes during SAVR. With Y-AAE, the most commonly used valve is size 29 valve, which has an opening matching the patient's native aortic annulus.^5^ We aimed to examine the perioperative outcomes, early survival, and follow-up hemodynamics of patients with native AV stenosis (AS) who underwent SAVR with Y-AAE and TAVR with the latest generation of TAVR valves (predominately self-expandable) during the same period. We hypothesized that by upsizing 3 to 4 valve sizes, SAVR + Y-AAE could provide better hemodynamics than TAVR in patients with AS.

## Methods

The Institutional Review Board of the University of Michigan (HUM00211344; January 10, 2022) approved the study protocol and publication of data. Patient written consent for the publication of the study data was waived because no patient information was revealed.

## Patient Selection

**Figure 1:**
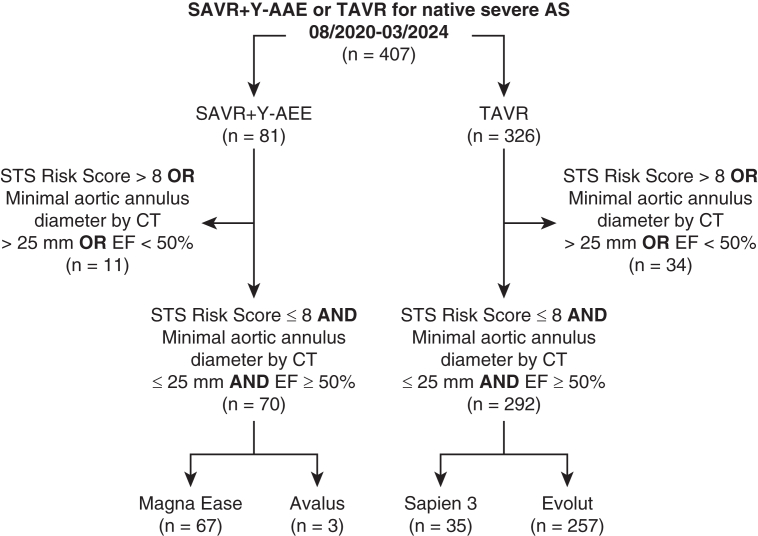
Flow chart of patient selection criteria. *SAVR*, Surgical aortic valve replacement; *Y-AAE*, Y-incision aortic annular enlargement; *TAVR*, transcatheter aortic valve replacement; *AS*, aortic stenosis; *STS*, Society of Thoracic Surgeons; *CT*, computed tomography; *EF*, ejection fraction. Magna Ease (Edwards Lifesciences). Avalus (Medtronic). Sapien 3 (Edwards Lifesciences). Evolut (Medtronic).

From August 2020 to March 2024, 326 patients with severe native AS, defined as effective orifice area (EOA) ≤1.0 cm^2^ or mean gradient (MG) ≥40 mm Hg, underwent TAVR, and 81 patients with severe native AS underwent SAVR with Y-AAE with bioprosthetic valves performed by a surgeon at the University of Michigan. Patients with a preoperative ejection fraction ≥50%, Society of Thoracic Surgeons (STS) predicted risk of mortality ≤8, and minimal aortic annular diameter ≤25 mm by computed tomography (CT) were included in the study: 70 patients in the SAVR with Y-AAE (SAVR + Y-AAE) group and 292 in the TAVR group. Patients in the SAVR + Y-AAE group received Magna Ease (Edwards Lifesciences) (n = 67) or Avalus valves (Medtronic) (n = 3), and patients in the TAVR group received Sapien 3 (Edwards Lifesciences) (n = 35) or Evolut (Medtronic) (n = 257) (Figure 1).

## Data Collection

Data from August 2020 to March 2024 was obtained from the STS database and supplemented with medical record review. Patients were followed with repeat transthoracic echocardiograms at our institution or outside institutions within 12 months, 12 to 24 months, and 24 to 36 months throughout a 36-month postoperative period. Left ventricular mass (LVM) was calculated using Devereux's formula from the American Society of Echocardiography and indexed to patient body surface area.^6^ In patients determined to have preoperative moderate or severe left ventricular hypertrophy (LVH) based on sex-specific thresholds of LVM index (LVMi) (≥109 g/m^2^ in women and ≥132 g/m^2^ in men), the LVM and LVMI were calculated within 12 months postintervention and again at 12 to 24 months postintervention. Prosthesis-patient mismatch (PPM) was defined by indexed EOA (iEOA) calculated from each patient's latest postoperative echocardiogram within the 36-month postoperative period using Valve Academic Research Consortium 3 (VARC 3) criteria.^7^ Mortality data were obtained from the Michigan Death Index, which was updated quarterly. The primary outcomes were hemodynamics during follow-up up to 36 months.

## Operative Technique

Details of the Y-AAE technique have been previously described and recently updated.^8^^,^^9^ Briefly, a Y-incision was made into the left-noncoronary commissure, extending from the aortotomy and to the aortomitral curtain. The incision was extended in a Y fashion underneath the aortic annulus to the left and right fibrous trigones. A 2 × 3 inch Hemashield patch (Getinge AB) was trimmed to 4 cm and sewn to the aortomitral curtain from the left and right fibrous trigones, and to the aortic annulus of left coronary and noncoronary sinuses. The prosthetic valve was sewn into the enlarged aortic root and tied down with 2-0 Ethibond (Ethicon) in a noneverting fashion. The aortotomy was closed with or without Roof technique.

## Statistics Analysis

**Figure E1:**
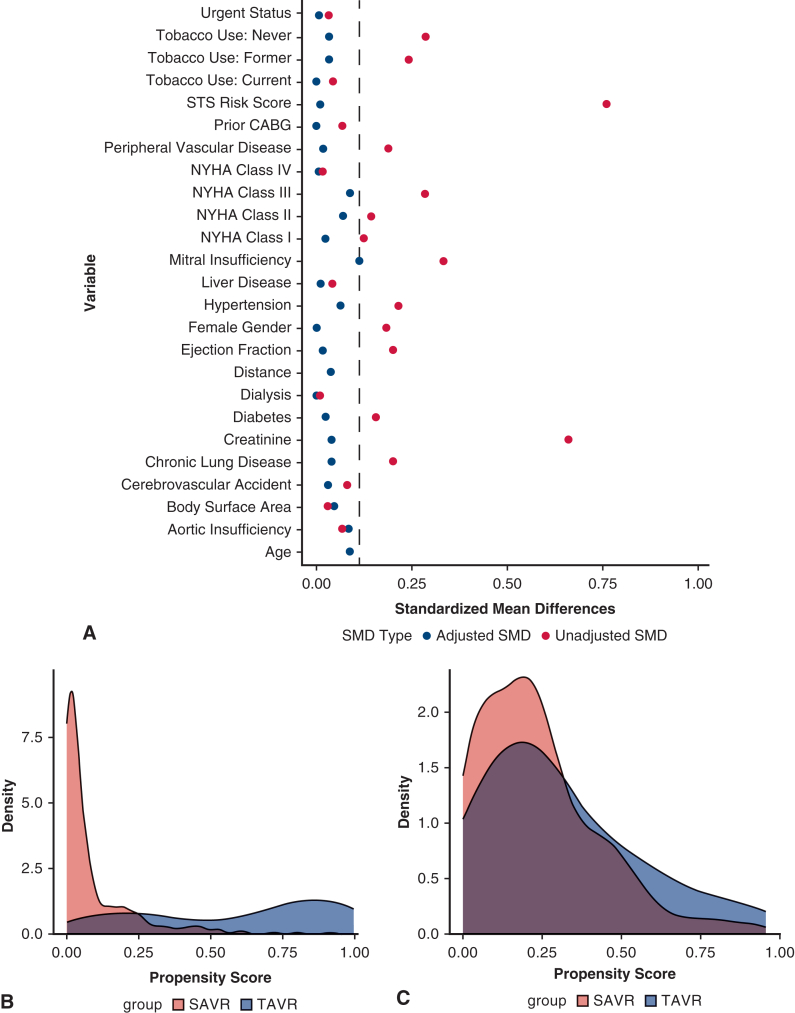
The propensity scores were derived using logistic regression with transcatheter aortic valve replacement (TAVR) versus surgical aortic valve replacement (SAVR) with aortic annular enlargement (AAE) as the dependent variable and covariates including age, sex, body surface area, cerebrovascular accident, chronic lung disease, creatinine, diabetes, dialysis, distance, ejection fraction, hypertension, liver disease, mitral insufficiency, New York Heart Association functional class, peripheral vascular disease, prior coronary artery bypass graft, Society of Thoracic Surgeons risk score, tobacco use (never, former, current), and status (urgent vs not). We performed 1:3 nearest-neighbor matching using a caliper width of 0.20 and greedy matching without replacement. Covariate balances were assessed using standardized mean differences, with values < 0.10 indicating acceptable balance. A Love plot of propensity score match (PSM) including all preoperative variables was generated (A) and indicated a well-balanced match with standard mean difference <0.1 (*black broken vertical line*) of all variables (see Table E1). Red dots: before PSM. Blue dots: after PSM. The propensity score distribution before matching (B), and after matching (C) showed that matching improved overlap between the TAVR and SAVR + Y-incision AAE groups, supporting successful balancing of covariates. *STS*, Society of Thoracic Surgeons; *CABG*, coronary artery bypass graft; *NYHA*, New York Heart Association; *SMD*, standardized mean difference.

Normality was assessed for all continuous variables. Nonparametric variables were presented as median (interquartile range [IQR]), whereas parametric variables were presented as mean (25%, 75%). Variables were then compared with Mann-Whitney *U* test (nonparametric) or Student *t* test (parametric). Categorical data were presented as n (%) and compared using χ^2^ tests. Kaplan-Meier methods were used to analyze early survival outcomes during 3-year follow-up. Multivariable Cox proportional hazard regression models were conducted using variables that were deemed potentially significant (*P* < .20) in a univariate Cox model, along with our treatment variable (SAVR + Y-AAE vs TAVR). Patients were then propensity score matched (PSM) based on all preoperative covariates (Figure E1, *A*-*C*). We conducted a 1:3 nearest-neighbor PSM between the TAVR and SAVR + Y-AAE patients using a standardized caliper width of 0.20. Univariate comparison tests and survival analyses were performed in the matched cohort as previously described. Data compilation and analysis were done with R version 3.6.2 (R Foundation for Statistical Computing), Excel (Microsoft), and SAS version 9.4 software (SAS Institute).

## Results

### Preoperative Demographic Characteristics

In the overall cohort, patients in the SAVR + Y-AAE group were younger (age 68 vs 78 years; *P* < .001), had more women (64% vs 51%; *P* = .06), fewer comorbidities, and lower STS scores (1.4% vs 2.7%; *P* < .001). Both groups had median STS scores in the low-risk range (Table 1). After PSM, there was no significant difference among all the preoperative variables between the 2 groups, and all standard mean differences were <0.1 (Table E1 and Figure E1, *A-C*).

**Table 1:** Preoperative demographic characteristics

Variable	Y-incision (n = 70)	TAVR (n = 292)	*P* value
Age (y)	68 (62, 73)	78 (73, 82)	<.001
Sex: Female	45 (64)	148 (51)	.06
Body surface area (cm^2^)	2.0 (1.8, 2.2)	2.0 (1.8, 2.2)	.75
Diabetes	19 (27)	118 (40)	.06
Renal failure on dialysis	0 (0)	2 (0.68)	>.99
Hypertension	51 (73)	275 (94)	<.001
Smoking status			<.001
Current Smoker	1 (1.4)	17 (5.8)	
Former smoker	20 (29)	154 (53)	
Never smoker	49 (70)	121 (41)	
Chronic lung disease	7 (10)	83 (28)	.002
Liver disease	2 (2.9)	20 (6.9)	.33
Peripheral vascular disease	2 (2.9)	53 (18.2)	.003
Cerebral vascular accident	2 (2.9)	30 (10.3)	.08
Preoperative creatinine (mg/dL)	0.9 (0.8, 1.0)	1.0 (0.8, 1.2)	.001
Prior coronary artery bypass grafting	1 (1.4)	22 (7.5)	.11
New York Heart Association functional class			<.001
I	12 (17)	4 (1.4)	
II	42 (60)	144 (50)	
III	12 (17)	130 (45)	
IV	4 (5.7)	14 (4.8)	
Ejection fraction (%)	60 (57, 65)	63 (59, 65)	.18
Aortic valve regurgitation			.92
None	19 (27)	75 (26)	
Trace/trivial	14 (20)	71 (24)	
Mild	24 (34)	113 (39)	
Moderate	11 (16)	27 (9.2)	
Severe	2 (2.9)	2 (0.7)	
Mitral valve regurgitation			<.001
None	17 (24)	21 (7.2)	
Trace/trivial	22 (31)	119 (41)	
Mild	24 (34)	103 (35)	
Moderate	6 (8.6)	38 (13)	
Severe	1 (1.4)	10 (3.4)	
Severe tricuspid valve regurgitation	0 (0)	3 (1.0)	>.99
Status			.48
Elective	68 (97)	275 (94)	
Urgent	2 (2.8)	17 (5.8)	
STS risk score	1.4 (0.9, 2.3)	2.7 (1.6, 4.2)	<.001

Values are presented as median (25%, 75%) for continuous data and n (%) for categorical data. *TAVR*, Transcathetetr aortic valve replacement; *STS*, Society of Thoracic Surgeons.

### Intraoperative Data

In the SAVR + Y-AAE group, the intraoperative median native aortic annulus was 21 mm (IQR, 19, 23 mm) measured by valve sizers and 24 mm (IQR, 22, 26 mm) derived from CT annulus area. All patients were upsized by a median of 3 (IQR, 3, 4) valve sizes. The median bioprosthetic valve size implanted was 29 (IQR, 27, 29). In the TAVR group, the median aortic annulus was 24 mm (IQR, 23, 26 mm) by annular area and 25 mm (IQR, 23, 26 mm) by perimeter by CT and the median valve sizes implanted were 26 (IQR, 25, 29) among the Sapien valves, and 29 (IQR, 29, 34) among the Evolut valves. The proportion of patients with small annuli, based on the Transcatheter Aortic Valve Replacement Versus Surgical Aortic Valve Replacement for Treating Elderly Patients With Severe Aortic Stenosis and Small Aortic Annuli (VIVA) and Self-Expanding or Balloon-Expandable TAVR in Patients with a Small Aortic Annulus (SMART) trial criteria, was similar between groups. In the SAVR + Y-AAE group, 43% of patients had planned concomitant procedures. Patients in the SAVR + Y-AAE group had more administration of intraoperative blood products than the TAVR group (30% vs 0.7%; *P* < .001) (Table 2). Intraoperative outcomes in the PSM cohort (Table E2) were similar to those in the overall cohort.

**Table 2:** Intraoperative data

Variable	Y-incision (n = 70)	TAVR (n = 292)	*P* value
Bicuspid aortic valve	27 (39)	33 (11)	<.001
Minimal aortic annulus by CT (mm)	21 (19, 23)	22 (20, 23)	.007
Aortic annulus derived from annular area by CT (mm)	24 (22, 26)	24 (23, 26)	.46
Aortic annulus derived from annulus perimeter by CT (mm)	25 (23, 36)	25 (23, 26)	.42
Aortic annulus by direct measurement (mm)	21 (19, 23)	–	–
Small annulus by VIVA trial∗	24 (34)	83 (28)	.42
Small annulus by SMART trial†	28 (40)	104 (36)	.56
No. of sizes enlarged	3 (3, 4)	–	–
Valve size implanted	29 (27, 29)	–	–
Edwards Sapien valve used	–	35 (12)	–
Edwards Sapien valve size	–	26 (25, 29)	–
Medtronic Evolut valve used	–	257 (88)	–
Medtronic Evolut valve size	–	29 (29, 34)	–
Crossclamp time (min)	136 (121, 159)	–	–
Cardiopulmonary bypass time (min)	169 (153, 198)	–	–
Planned concomitant procedures			
Mitral valve repair/replacement	1 (1.4)	–	–
CABG	9 (13)	–	–
Maze/left atrial appendage excision	10 (14)	–	–
Ascending aortic procedure	10 (14)	–	–
Isolated AVR + AAE§	40 (57)	–	–
Crossclamp time (min)	130 (117, 141)		
Cardiopulmonary bypass time (min)	156 (142, 172)		
Intraoperative blood products	21 (30)	2 (0.7)	<.001
Packed red blood cells (U)‡	0.0 (0.0, 2.0)	1.0 (0.0, 2.0)	.46
Platelets‡	2.0 (1.0, 2.0)	0.0 (0.0, 1.0)	.04
FFP‡	1.0 (0.0, 2.0)	0.0 (0.0, 0.5)	.11
Cryoprecipitate‡	0.0 (0.0, 1.0)	0.0 (0.0, 0.0)	.13

Values are presented as median (25%, 75%) for continuous data and n (%) for categorical data. *TAVR*, Transcatheter aortic valve replacement; *CT*, computed tomography; *VIVA*, Transcatheter Aortic Valve Replacement Versus Surgical Aortic Valve Replacement for Treating Elderly Patients With Severe Aortic Stenosis and Small Aortic Annuli; *SMART*, Self-Expanding or Balloon-Expandable TAVR in Patients with a Small Aortic Annulus; *CABG*, coronary artery bypass graft; *AVR*, aortic valve replacement; *AAE*, aortic annular enlargement; *FFP*, fresh frozen plasma.

∗ Defined as a minimal aortic annular diameter ≤21.5 mm and a mean aortic annular diameter <23 mm.^6^

† Defined as an aortic annular area ≤430 mm^2^.^10^

‡ Includes only patients who received any type of intraoperative blood products.

§ Both first time and reoperative AVR after prior cardiac surgeries were included.

### Perioperative Outcomes

Perioperatively, there was no significant difference in operative mortality, stroke, and new renal failure requiring dialysis, between the 2 groups (Table 3). The rate of complete heart block requiring pacemaker implantation (1.4% vs 10.3%; *P* = .03) and new-onset left bundle branch block were significantly lower in the SAVR + Y-AAE group compared with the TAVR group (Table 3). Perioperative outcomes in the PSM cohort were similar to those in the overall cohort with the exception of pacemaker implantation (3.1% vs 7.7%; *P* = .65) (Table E3).

**Table 3:** Perioperative data

Variable	Y-incision (n = 70)	TAVR (n = 292)	*P* value∗
Atrial fibrillation	22 (31)	14 (4.8)	<.001
Atrial fibrillation at discharge	2 (2.8)	10 (3.4)	>.99
Complete heart block requiring pacemaker	1 (1.4)	30 (10.3)	.03
New, nontransient left bundle branch block†	5 (7.1)	59 (20)	.01
Stroke	0 (0)	9 (3.1)	.29
Deep sternal infection	0 (0)	–	–
Reoperation for bleeding	1 (1.4)	0 (0)	.44
Prolonged ventilation	3 (4.3)	2 (0.7)	.08
Pneumonia	3 (4.3)	2 (0.7)	.08
New renal failure requiring dialysis	2 (2.9)	2 (0.7)	.36
Postoperative length of stay	7 (6.0, 9.0)	1.0 (1.0, 2.0)	<.001^a^
Readmission for cardiac reasons‡	1 (2.6)	28 (9.6)	<.001^b^
Operative mortality§	0 (0)	2 (0.7)	>.99^a^

Values are presented as median (25%, 75%) for continuous data and n (%) for categorical data. *TAVR*, Transcatheter aortic valve replacement.

∗ Superscripts (a, b) in the *P* values column indicate (a) Mann-Whitney U test and (b) ꭓ^2^ test.

† Nontransient defined as new-onset left bundle branch block postoperatively lasting >30 days.

‡ Readmission for cardiac reasons at 30 days.

§ Operative mortality was defined as death within hospital admission or 30 days of discharge.

### Follow-up Echocardiography Results

**Figure 2:**
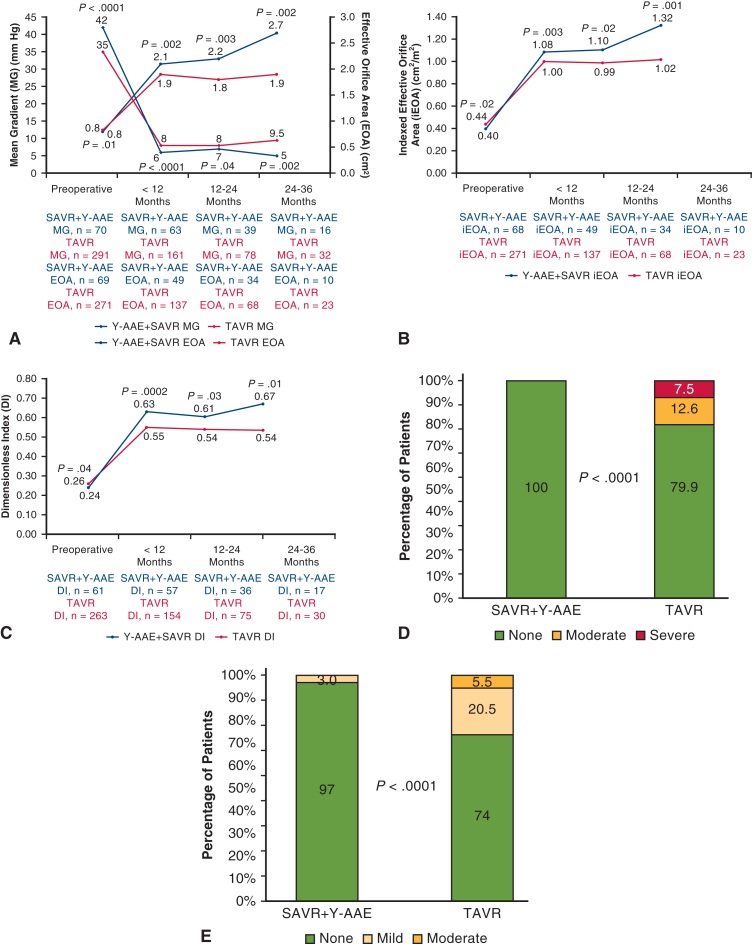
Preoperatively, the surgical aortic valve replacement (SAVR) with Y-incision aortic annular enlargement (Y-AAE) group had significantly higher mean gradient (MG) across the aortic valve (42 vs 35 mm Hg; *P* < .0001). A, At <12, 12 to 24, and 24 to 36 months postoperatively, the SAVR + Y-AAE group had a significantly lower MG across the prosthetic valve, larger effective orifice area (EOA) (A) and indexed EOA (iEOA) (B), greater dimensionless index (DI) (C), lower rate of moderate/severe prothesis-patient mismatch (0% vs 20.1%; *P* < .0001) (D), and lower incidence (3.0% vs 26%; *P* < .0001) and severity of aortic insufficiency (3.0% mild only vs 20.5% mild, and 5.5% moderate) (E) than the transcatheter aortic valve replacement (TAVR) group.

**Figure E2:**
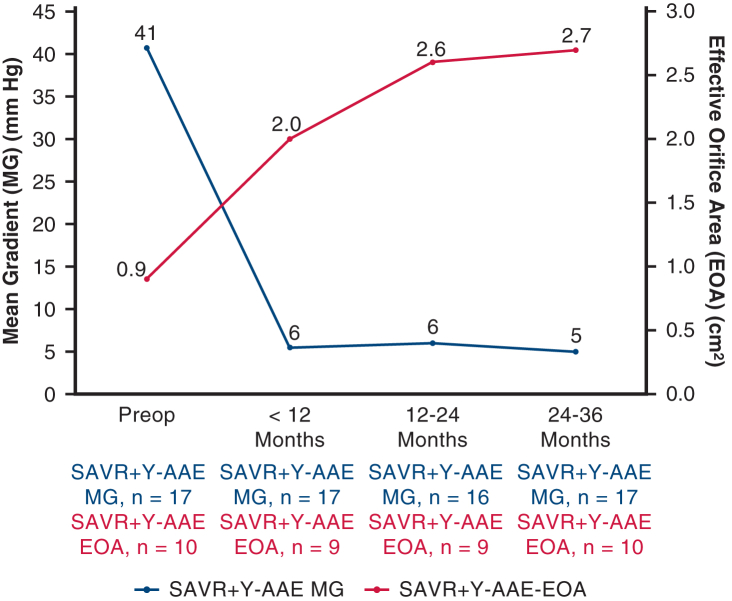
Only patients who had follow-up echocardiograms up to 24 to 36 months were included. The patients' effective orifice area (EOA) continued to improve to 2.7 cm^2^ at 24 to 36 months compared with 0.9 cm^2^ at preoperative (preop) (*P* < .001), 2.0 cm^2^ at <12 months (*P* = .06), and 2.6 cm^2^ at 12 to 24 months (*P* = .44). The mean gradient (MG) was significantly lower at 24 to 36 months than the preoperative mean gradient (5 vs 41 mm Hg; *P* < .0001) and remained consistent up to 24 to 36 months. Values are presented as median. *SAVR*, Surgical aortic valve replacement; *Y-AAE*, Y-incision aortic annular enlargement.

Repeat postoperative echocardiograms showed significantly lower aortic valve MG, larger EOA, iEOA, and dimensionless index (DI) in the SAVR + Y-AAE group compared with the TAVR group at the <12-, 12- to 24-, and 24- to 36-month postoperative periods (Figure 2, *A-C*). The median MG was 5 versus 9.5 mm Hg (*P* < .01) and the median EOA was 2.7 versus 1.9 cm^2^ (*P* < .01) at 24 to 36 months postoperatively (Figure 2, *A*). The continuous improvement of EOA and MG remained the same in the subgroup of patients who had complete echocardiography follow-up up to 24 to 36 months (Figure E2). The rate of moderate/severe PPM was significantly lower in the SAVR + Y-AAE group than in the TAVR group (0% vs 20.1%; *P* < .001) (Figure 2, *D*). The incidence (3.0% vs 26%; *P* < .001) and severity (3.0% mild vs 20.5% mild and 5.5% moderate) of aortic regurgitation and paravalvular leak were significantly lower in the SAVR + Y-AAE group than in the TAVR group (Figure 2, *E*).

**Figure 3:**
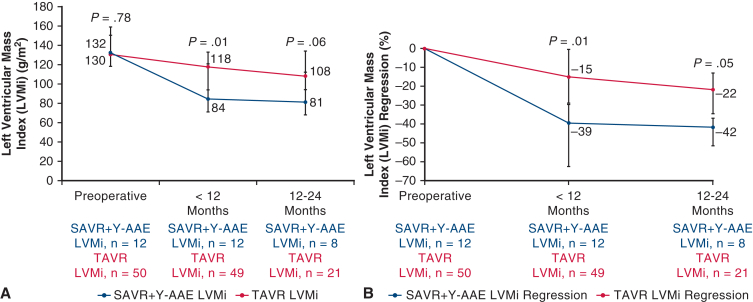
The preoperative left ventricular mass index (LVMi) in patients with moderate/severe left ventricular hypertrophy was similar between the surgical aortic valve replacement (SAVR) with Y-incision aortic annular enlargement (Y-AAE) and transcatheter aortic valve replacement (TAVR) groups (A) (132 g/m^2^; interquartile range [IQR], 118, 150 g/m^2^ vs 130 g/m^2^; IQR, 118, 158 g/m^2^; *P* = .78), and was significantly lower in the SAVR + Y-AAE group at 12 months (84 g/m^2^; IQR, 71, 120 g/m^2^ vs 118 g/m^2^; IQR, 94, 133 g/m^2^; *P* = .01) (B). The LVMi regression percentage was also significantly greater in SAVR + Y-AAE than that in TAVR group, at 12 months (39%; IQR, 16%, 49% vs 15%; IQR, 1.5%, 30%; *P* = .01) and 12 to 24 months (42%; IQR, 32%, 47% vs 22%; IQR, 9%, 31%; *P* = .05). Bars represent IQR.

**Figure E3:**
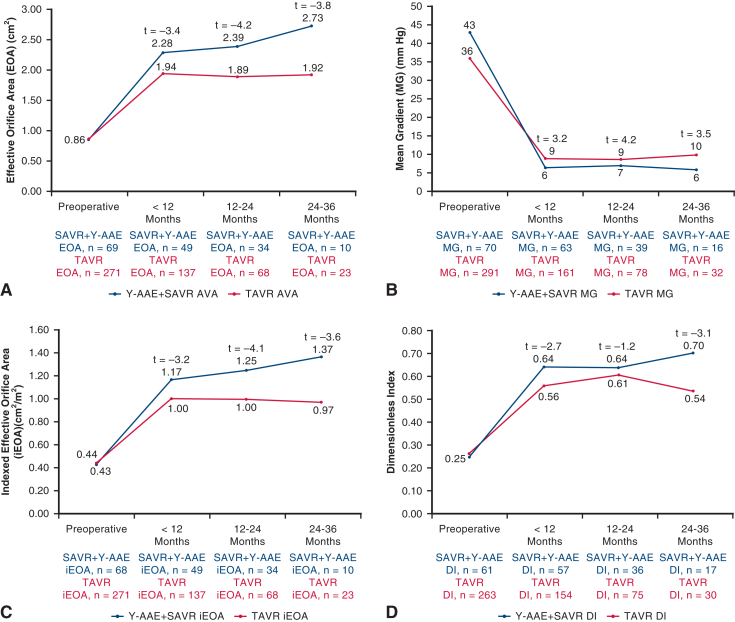
Including time as a factor on the hemodynamic outcomes, the mixed effects model confirmed significantly larger effective orifice area (EOA) (A), lower mean aortic valve gradients (B), larger indexed EOA (iEOA) (C), and a better dimensionless index (DI) over time (D) in the transcatheter aortic valve replacement (TAVR) versus the surgical aortic valve replacement (SAVR) with Y-incision aortic annular enlargement (Y-AAE) group compared with the transcatheter aortic valve replacement (TAVR) group in the overall cohort. T < −2.0 or > 2.0 was considered significant between SAVR + Y-AAE and TAVR groups. *MG*, Mean gradient.

Finally, the preoperative LVMi among patients with moderate to severe LVH showed no significant differences between the groups, (132 vs 130 g/m^2^; *P* = .78) but was significantly lower in the SAVR + Y-AAE group at 12 months (84 vs 118 g/m^2^; *P* = .01) and trended lower at 12 to 24 months (81 vs 108 g/m^2^; *P* = .062). The LVMi regression was also greater in the SAVR + Y-AAE group at 12 months, (39% vs 15%; *P* = .01) and 12 to 24 months (42% vs 22%; *P* = .05) (Figure 3, *A* and *B*). The mixed effects model, created as a sensitivity analysis, showed results consistent with the primary analysis, confirming significantly lower mean AV gradients, larger EOA and iEOA, and improved DI in the SAVR + Y-AAE group compared with the TAVR group (Figure E3, *A-D*).

### Readmission and Early Survival

**Figure 4:**
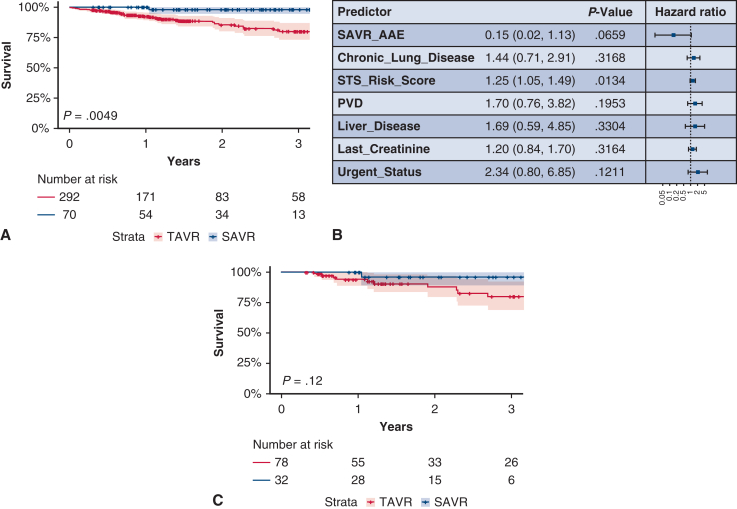
A, In the overall cohort, the 3-year survival was significantly better in the surgical aortic valve replacement (SAVR) with Y-incision aortic annular enlargement (Y-AAE) group (98%; 95% CI, 94-99) compared with the transcatheter aortic valve replacement (TAVR) group (79%; 95% CI, 73-87) (*P* < .01). B, The hazard ratio of SAVR + Y AAE for early mortality was 0.15 (95% CI, 0.02-1.13) (*P* = .066). C, In the matched cohort, the 3-year survival in the SAVR-Y + AAE group was 96% (95% CI, 89-99) versus 79% (95% CI, 67-92) in the TAVR group (*P* = .12). *STS*, Society of Thoracic Surgeons; *PVD*, peripheral vascular disease.

The rate of readmission for cardiac reasons at 30 days was significantly lower in the SAVR + Y-AAE group than the TAVR group (2.6% vs 9.6%; *P* < .001). The 3-year survival was 98% (95% CI, 94%-99%) in the SAVR + Y-AAE group compared with 79% (95% CI, 73%-87%) in the TAVR group (*P* < .01) (Figure 4, *A*). After univariable Cox model analysis (Table E4) to select variables for multivariable analysis, the hazard ratio of SAVR + Y AAE for early mortality was 0.15 (95% CI, 0.02-1.13; *P* = .066) (Figure 4, *B*). After PSM, the 3-year survival was 96% in the SAVR + Y-AAE group versus 79% in the TAVR group (*P* = .12) (Figure 4, *C*).

## Comment

In this study, we reported SAVR+Y-AAE demonstrated significantly better hemodynamics, lower PPM rates, and greater LVMi regression than TAVR in low- to intermediate-risk patients with native AS. Patients treated with SAVR + Y-AAE had similar operative mortality and demonstrated lower rates of postoperative complete heart block requiring pacemaker implantation and aortic insufficiency at 3 years than those treated with TAVR.

Given that the mean aortic annular diameter is 21 mm in women and 23 mm in men,^11^ it is unsurprising that the most-used valve sizes among the TAVR versus SAVR trials and large SAVR series are 21 and 23.^1^^,^^3^^,^^12^ Because of the sewing ring and struts of the SAVR valve, the prosthetic valve's opening is approximately 5 to 7 mm smaller than its labeled size.^5^^,^^12^ Implanting a valve size equivalent to the measured annular diameter reduces the open valve area by approximately 50% to 60%.^5^^,^^13^ Because TAVR valves do not include struts and sewing rings, and physicians tend to choose larger TAVR valves if allowed, it is unsurprising that recent trials report that TAVR valves (particularly self-expanding designs) elicit better hemodynamics than patients treated with SAVR. In these same trials, 4.6% or fewer patients^1^ underwent AAE in the SAVR groups. As we previously reported, the Y-AAE can upsize the prosthetic valve by 3 to 4 valve sizes, allowing implantation of a much larger prosthetic valve with the valve opening equivalent to or larger than the patient's native aortic annulus.^5^^,^^8^^,^^9^ Patients treated with SAVR + Y-AAE had a median postoperative AV area of 2.7 cm^2^ at 24 to 36 months and demonstrated continuous improvement throughout the 36-month period (Figure 2, *A*, and Figure E2), likely due to improved flow and increasing diameter of the LV outflow tract^7^ from the regression of LVH (Figure 3, *A* and *B*). Previous studies demonstrated that patients with valve areas of 2.5 cm^2^ and 1.5 cm^2^ have similar mean gradients across the prosthetic valve at rest. However, exercise elicits a 50-mm Hg difference in prosthetic valve MG between the two AV areas.^14^ Given these results, it is important to consider that echocardiography performed at rest may not illuminate imbalance in hemodynamic performance between valves. Our study reported a significant improvement in EOA, MG, and DI. The large EOA in the SAVR + Y-AAE group would significantly improve hemodynamics during exercise and improve quality of life. EOA also has a significant influence on prosthetic valve longevity and need for reintervention.^15^ The main mechanism of structural deterioration in bovine pericardial valves is calcification and stenosis. Implanting a SAVR valve with a large opening (2.7 cm^2^) has the potential to maintain an EOA longer than a TAVR valve with a smaller opening (1.9 cm^2^) and increase time before the prosthetic valve becomes severely stenotic.^14^ In our study, no SAVR + Y-AAE patients required reintervention for valvular degeneration, paravalvular leak, or other cardiac complications except 1 patient requiring reoperation for endocarditis at 6 months after the initial operation.

Low-risk patients tend to live longer and will likely require future AV reintervention. Implanting a large valve size for first-time intervention is a key factor in avoiding PPM and fast AV structural deterioration, and improving potential for future valve-in-valve TAVR. With Y-AAE, the median implanted valve size was size 29 (Table 2) and resulted in no PPM (Figure 2, *D*). With a larger opening and no PPM, the prosthetic valve has the potential to provide greater longevity. Although small prosthetic valves (19-23 mm) pose a challenge to future valve-in-valve TAVR, large prosthetic valves (27-29 mm) pose no such challenge. Based on postoperative CT angiography data, the prosthetic valve is sitting at the natural AV position without tilting.^6^^,^^16^ Patients treated with SAVR + Y-AAE have a valve-to-coronary distance range of 4.9 to 6.6 mm, valve-to-aorta distance ranges 4.4 to 8.1 cm, median sinus of Valsalva diameter of 38 mm, and sinotubular junction of 36 to 40 mm.^16^ These measurements of aortic root reconstruction make patients treated with Y-AAE good candidates for future valve-in-valve TAVR. The TAVR-in-SAVR procedure is already an effective and safe treatment for structural valve deterioration.^17^ By implanting a large valve in first-time intervention, the EOA provides an optimal candidacy for future valve-in-valve hemodynamics and survival. The longevity of TAVR valves remains unestablished, and low-risk patients treated with TAVR for first-time intervention may require second and third procedures depending on their lifespan. When these patients are not TAVR-in-TAVR candidates after a first or second TAVR, they will eventually need SAVR. A recent study of propensity score-matched patients with AS reported those treated with TAVR-SAVR had an 11.3% operative morality rate.^18^ Our results support SAVR + Y-AAE as the first intervention in patients who are surgical candidates.

After PSM, the 3-year survival in SAVR + Y-AAE was 96% versus 79% in TAVR (*P* = .12) (Figure 4, *C*) and hazard ratio of SAVR + Y-AAE was 0.15 in the overall cohort (*P* = .066) (Figure 4, *B*). We postulate that given a larger sample size and longer follow-up, this difference may reach significance. The SAVR + Y-AAE group had a very low pacemaker rate, minimal aortic insufficiency, no PPM, continuously improving hemodynamics, and increased LVMi regression; therefore, we expect patients treated with SAVR + Y-AAE would report favorable survival in longer follow-up. As previously established, aortic insufficiency, moderate/severe PPM, and LVM regression are independently associated with long-term survival.^19^

Our study is limited as a single-center, retrospective analysis conducted with a small sample size. The TAVR and SAVR patient populations were not the same and PSM may not completely overcome the difference. However, the 2 groups were comparable for the comparison of hemodynamics because the annular size of the 2 groups was very similar and only low-to intermediate-risk patients with ejection fraction ≥50% were included. By upsizing 3 to 4 valve sizes, it was understandable that the SAVR + Y-AAE group had better hemodynamics than the TAVR group, in whom the aortic annulus/root was not enlarged. The number of patients with the echocardiogram measurements at 24 to 36 months was limited. These results came from a high-volume valve center and may not be generalizable. The SAVR + Y-AAE group was from a single surgeon's experience because other surgeons performed AAE differently or had no follow-up echocardiogram.

## Conclusions

**Figure 5:**
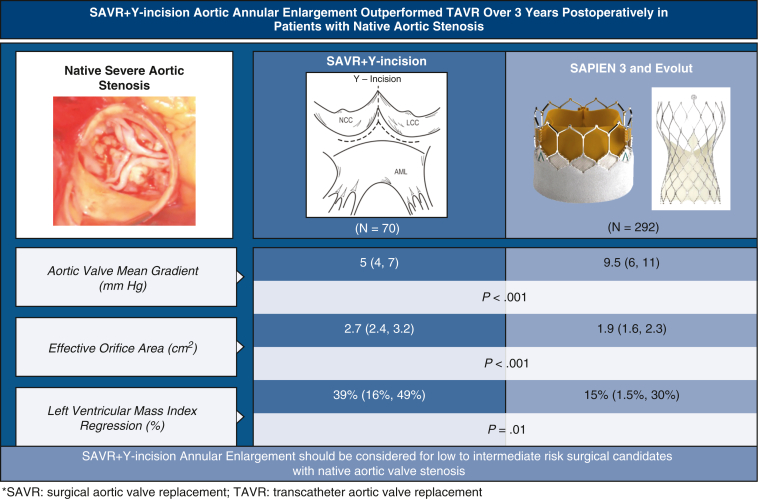
Graphical abstract. SAVR + Y-incision Aortic Annular enlargement achieved significantly better hemodynamics and regression left ventricle hypertrophy than TAVR, and should be considered for low to intermediate risk patients with native aortic valve stenosis. *SAVR*, Surgical aortic valve replacement; *TAVR*, transcatheter aortic valve replacement. Sapien 3 (Edwards Lifesciences). Evolut (Medtronic).

Low-to intermediate-risk patients with severe native AS should be considered for SAVR + Y-AAE for its excellent hemodynamics and early outcomes (Figure 5). This study calls for prospective randomized trials of SAVR + Y-AAE versus TAVR.

## Conflict of Interest Statement

Dr Yang reports relationships with Medtronic, Edwards, Corcym, Balance, and Ethicon Endo-Surgery. All other authors reported no conflicts of interest.

The *Journal* policy requires editors and reviewers to disclose conflicts of interest and to decline handling or reviewing manuscripts for which they may have a conflict of interest. The editors and reviewers of this article have no conflicts of interest.

## Figures and Tables

**Table E1:** Preoperative demographic charactetristics

Variable	Y-incision matched (n = 32)	TAVR matched (n = 78)	SMD value
Age	71 (67, 73)	72 (68, 78)	.08
Sex: Female	12 (38)	30 (38)	<.01
BSA (cm^2^)	2.0 (1.7, 2.3)	2.0 (1.8, 2.2)	.04
Diabetes	8 (25)	22 (28)	.02
Renal failure on dialysis	0 (0)	2 (0.68)	<.01
Hypertension	28 (88)	68 (87)	.06
Smoking status			
Current smoker	1 (3.1)	3 (3.9)	<.01
Former smoker	12 (38)	34 (44)	.03
Never smoker	19 (59)	41 (53)	.03
Chronic lung disease	7 (22)	13 (17)	.04
Liver disease	1 (3.1)	2 (2.6)	.01
Peripheral vascular disease	1 (3.1)	4 (5.1)	.02
Cerebral vascular accident	2 (6.3)	3 (3.9)	.03
Preoperative creatinine (mg/dL)	0.8 (0.8, 1.0)	0.9 (0.8, 1.0)	.03
Prior coronary artery bypass	1 (3.1)	3 (3.9)	<.01
New York Heart Association functional class			
I	2 (6.3)	2 (2.6)	.03
II	19 (60)	41 (53)	.06
III	9 (28)	30 (38)	.08
IV	2 (6.3)	5 (6.4)	.005
Ejection fraction (%)	60 (58, 65)	62 (59, 65)	.02
Aortic valve regurgitation	20 (63)	54 (69)	.07
Mitral valve regurgitation	27 (84)	71 (91)	.09
Status			
Urgent	2 (6.2)	4 (5.1)	.005
STS risk score	1.7 (1.08, 3.0)	2.0 (1.4, 2.7)	.008

Values are presented as median (25%, 75%) for continuous data and n (%) for categorical data. *TAVR*, Transcatheter aortic valve replacement; *SMD*, standardized mean difference; *BSA*, body surface area; *STS*, Society of Thoracic Surgeons.

**Table E2:** Intraoperative data

Variable	Y-incision matched (n = 32)	TAVR matched (n = 78)	*P* value
Bicuspid aortic valve	9 (28)	6 (7.7)	.01∗
Minimal native aortic annulus by CT (mm)	20 (19, 23)	21 (20, 23)	.01†
Direct measurement intraoperative aortic annulus (mm)	21 (19, 23)	–	–
Small annulus by VIVA trial‡	15 (47)	27 (35)	.32∗
Small annulus by SMART trial§	16 (50)	32 (41)	.52∗
No. of sizes enlarged	3 (3, 4)	–	–
Valve size implanted	28 (27, 29)	–	–
Edwards Sapien valve used	–	7 (9.0)	–
Edwards Sapien valve size	–	26 (25, 26)	–
Medtronic Evolut valve used	–	71 (91)	–
Medtronic Evolut valve size	–	29 (29, 34)	–
Crossclamp time (min)	143 (123, 169)	–	–
Cardiopulmonary bypass time (min)	174 (159, 213)	–	–
Planned concomitant procedures			
Mitral valve repair/replacement	1 (3.1)	–	–
Concomitant CABG	5 (16)	–	–
Maze/left atrial Appendage ligation	7 (22)	–	–
Ascending aortic procedure	2 (6.3)	–	–
Isolated AVR + AAE¶	17 (53)	–	–
Crossclamp time (min)	136 (127, 145)		
Cardiopulmonary bypass time (min)	159 (145, 170)		
Intraoperative blood products	21 (30)	2 (0.7)	<.0001∗
Packed red blood cells (U)‖	1.0 (0.0, 2.0)	0.0 (0.0, 0.0)	.45†
Platelets‖	2.0 (1.0, 2.0)	0.0 (0.0, 0.0)	.28†
FFP‖	1.0 (0.0, 2.0)	2.0 (2.0, 2.0)	.44†
Cryoprecipitate‖	0.0 (0.0, 1.0)	0.0 (0.0, 0.0)	.55†

Values are presented as median (25%, 75%) for continuous data and n (%) for categorical data. *TAVR*, Transcatheter aortic valve replacement; *CT*, computed tomography; *VIVIA*, Transcatheter Aortic Valve Replacement Versus Surgical Aortic Valve Replacement for Treating Elderly Patients With Severe Aortic Stenosis and Small Aortic Annuli; *SMART*, Self-Expanding or Balloon-Expandable TAVR in Patients with a Small Aortic Annulus; *CABG*, coronary artery bypass graft; *AVR*, aortic valve replacement; *AAE*, aortic annular enlargement; *FFP*, fresh frozen plasma.

∗ χ^2^ test.

† Mann-Whitney *U* test.

‡ Small annulus by VIVA trial was defined as a minimal aortic annular diameter ≤21.5 mm and a mean aortic annular diameter <23 mm.^8^

§ Small annulus by SMART trial was defined as an aortic annular area ≤430 mm^2^.^19^

‖ Includes only patients who received any type of intraoperative blood products.

¶ Both first time and reoperative AVR after prior cardiac surgeries were included.

**Table E3:** Perioperative data

Variable	Y-incision matched (n = 32)	TAVR matched (n = 78)	*P* value
Atrial fibrillation	10 (31)	4 (5.1)	<.01∗
Atrial fibrillation at discharge	1 (3.1)	3 (3.9)	>.99∗
CHB requiring pacemaker	1 (3.1)	6 (7.7)	.65∗
Stroke	0 (0)	2 (2.6)	.89∗
Deep sternal infection	0 (0)	0 (0)	–
Reoperation for bleeding	0 (0)	0 (0)	–
Prolonged ventilation	1 (3.1)	1 (1.3)	1.0∗
Pneumonia	1 (3.1)	0 (0.0)	.64∗
New renal failure requiring dialysis	1 (3.1)	0 (0.0)	.64∗
Postoperative length of stay	8 (6.0, 13)	1.0 (1.0, 2.0)	<.0001†
Readmission for cardiac reasons	0 (0.0)	7 (9.0)	<.0001∗
Operative mortality‡	0 (0.0)	0 (0.0)	>.99∗

Values are presented as median (25%, 75%) for continuous data and n (%) for categorical data. *TAVR*, Transcatheter aortic valve replacement; *CHB*, complete heart block.

∗ ꭓ^2^ test.

† Mann-Whitney *U* test.

‡ Operative mortality was defined as death within hospital admission or within 30 days of discharge.

**Table E4:** Univariable Cox models

Variable	Hazard ratio	Lower CI	Upper CI	*P* value
Age	1.012893	0.975039	1.052218	.509756
Chronic lung disease	1.823254	0.904453	3.675429	.093113
Gender	1.24132	0.6245	2.467373	.537399
STS risk score	1.348799	1.14813	1.58454	.000272
PVD	2.043512	0.925681	4.511213	.076923
BMI	1.006879	0.962818	1.052957	.763963
Diabetes	1.157115	0.587597	2.278628	.672969
Dialysis	3.03E-07	0	Inf	.997385
Hypertension	1.450348	0.443203	4.746154	.538767
Smoking status	1.695324	0.225558	12.74229	.607998
Liver disease	2.23647	0.788055	6.347021	.13043
Preoperative creatinine	1.330785	1.017684	1.740214	.036794
Prior valve	8.24E-07	0	∞	.997308
EF	1.032159	0.976873	1.090574	.259776
BSA	0.794434	0.235427	2.680765	.710749
Prior CABG	0.541322	0.073927	3.963764	.545715
Status	2.964295	1.04412	8.415741	.041245

*STS*, Society of Thoracic Surgeons; *PVD*, peripheral vascular disease; *BMI*, body mass index; *EF*, ejection fraction; *BSA*, body surface area; *CABG*, coronary artery bypass graft.
